# Case of Hydrophobia, Treated with Superacetate of Lead

**Published:** 1828-07

**Authors:** Charles E. Jenkins


### *Case of Hydrophobia, treated with Superacetate of Lead.*

By CHARLES E. JENKINS.

I BEG leave to offer to your notice a case of hydrophobia, which, as far as my recollection extends, is the severest on record: the disease, in its whole progress from the first appearance of the symptoms, not occupying a greater space of time than thirty hours and a half.

33, *Great Prescot-street.*

WILLIAM THOMAS HAZLAM, a fine young man, aged twenty-two years, residing at No. 8, Jones'-building, Gower's-walk, Whitechapel, by occupation a labourer, had complained, for three months previous to his fatal malady, of heaviness in the head, weariness, and constant propensity to sleep, which he attributed to having caught cold.

On Saturday, November 4th, in the afternoon, he complained of being ill, refused to take his tea; and, at thirty minutes past eleven o'clock, first discovered an inability to swallow fluids, which he ascribed to flatulence, which, meeting the descending liquid, would not allow it to pass into the stomach.

He continued getting worse in this respect until eleven A.M. November 5th, when I first saw him. I then found him dressed, sitting by the fire: he complained of wind in the stomach, and lamented his incapability of swallowing liquids. In reply to the question, whether he felt pain, he said, "I am in no pain whatever; I can eat a crust of bread as well as any one in the room, but I can't drink: my throat is not sore, and I don't know why I can't swallow." Pulse eighty; tongue furred, dry and parched; bowels regular; skin moist.

Two P.M.—Now the peculiar symptom, before only suspected, showed itself in all its characteristic intensity: on the instant of his attempting to take any liquid, to which he was constantly incited by the most tormenting thirst, a frightful convulsion of the whole body succeeded. He was now in bed, and had rolled the bedclothes round him, and girted them tight round his neck. The least breath of air, the undulation of the bedclothes (unless caused by himself), or the slightest thing in motion, produced the utmost agony and terror.

Four P.M.—Being considerably worse, Dr. T. DAVIES, of New Broad-street, was called in at the patient's request, who recommended (the exhibition of liquid medicine being impossible,) a trial of the Plumbi Superacetas in substance, in doses of half a grain every half hour: this the patient managed pretty well, by placing the powder on his tongue himself, but he would not suffer any other person to attempt it for him; and even this simple method of receiving the medicine was followed on every occasion by a slight paroxysm, as soon as the powder touched his tongue.

Six P.M.—The disease greatly aggravated. Pulse 130; mouth still parched; thirst incessant, beyond any thing I ever witnessed before: he continually cried "Oh! that I could drink quarts." His countenance expressed fear and horror, combined sometimes with fury, at others with the saddest melancholy.

Nine P.M.—Found him sitting on the bed, supported by two attendants, of whose breathing on him he continually complained. He would not suffer the candle to approach him, or to be moved. The sight of a sheet of white paper distressed him exceedingly. A small mirror being shown him, threw him into strong convulsions: on his recovery, being questioned as to the cause of them, he replied "Because my face looks so white, I can't bear it."*

He had drilled his two attendants into a sort of military observance, and this was the order of it: they holding him under each arm, the one held a teaspoon containing a little milk, which, when the patient gave the word "Now," he endeavoured to throw into his mouth; but the moment it approached his lips, he threw himself backward on the bed in the utmost agony of convulsion, which lasted generally twenty seconds. A tranquil interval of ten seconds succeeded; then suddenly and furiously he would cry out "Ready!" which meant that they were to raise him up again. After a few seconds, in which he strained his courage to another dreadful effort, the word "Now" would be repeated, the spoon again approach his lips, and the convulsions as certainly follow. This process continued without intermission or variation until four o'clock. In one of his tranquil moments, he said "I don't know what you think of it, but I call this killing work."

Eleven P.M.—His mouth, which had throughout continued parched, began to discharge saliva in abundance, which he blew forth with convulsive energy. He showed a mischievous inclination to blow his saliva upon the persons present: this he would apologise for in his calm intervals, and, with a bitter smile, would request them to "keep out of the way next time."

A tendency to vomit, perhaps occasioned by the Plumbi Superacetas, disturbed him; spasmodic action of the abdominal muscles also frequently occurred.

It was a distressing operation to him to wipe off the saliva which collected about his mouth, and annoyed him very much. After

* His face at this time was flushed.

many fruitless attempts, he would pass his handkerchief over his lips with the rapidity of lightning, but the convulsions, ever at hand, still more swift, would, at the slightest contact, inevitably follow.

A small portion of wine, not exceeding the third part of a tea-spoonful, having, in the only felicitous attempt I witnessed, passed into his mouth, elicited from him loud exclamations of joy : "Oh ! (said he,) how that drop of wine has comforted me! I shall be cured now; I feel myself a new man!" But this calm was of only three minutes' duration ; and the longest interval he enjoyed was succeeded by a fearful exacerbation. Pulse 140, very small; body drenched in constant perspiration; face red and swollen.

This state of things continued, the paroxysms equally distinct and frequent, but weaker, until four o'clock, when all convulsions ceased, and he died about five o'clock, so easily that his attendants knew not the precise moment of his death.

A very strict inquiry was made by Dr. Davies and myself into all the circumstances of his life. His father and mother declared he had never been bitten by any animal during childhood; and he as strenuously denied it on his own part, and continued to do so until two hours before his death, when he confessed to his father that a dog kept by his master had, nine months before, bitten him in the instep, thigh, and hand. Being questioned as to the cause of his obstinate denial of the fact, he said, "If I had told those Doctors the truth, they would have caused me to be put to death."

This was not the language of unhinged reason, but a mere expression of a vulgar opinion, which obtains with some, that a hydrophobic patient may be with impunity destroyed.

During the whole progress of the disease, he felt no pain ; his reason never deserted him; he had a craving appetite, but, notwithstanding his assertion that he could eat a crust of bread, I never found that he could suffer any thing to pass his lips: his whole frame, like a highly charged electrical battery, required but a touch to be thrown into sudden and violent action.

Distant sounds did not affect him. In the room he would not allow conversation, though carried on in the lowest whisper. All white and shining bodies were held by him in utter abhorrence.

I have not been able to discover whether the dog had ever exhibited any rabid appearances: he is dead, having been starved by the neglect of the deceased, who had the charge of feeding him.

*Examination of the body.*—Externally: discovered a scar on the inner part of the left leg, corresponding with his statement to his father of one of the bites of the dog, across which branches of the saphena nerve extended themselves. The nerve presented no remarkable appearance.

Brain: Dura mater very adherent. Veins of the surface very much distended. More bloody points than usual in cutting the medullary substance. Ventricles nothing remarkable. Plexus choroides unusually pale. Serous fluid at the basis about two drachms. Under the tongue, nothing unusual.

Larynx and trachea red; fine vessels, in arborescent forms, extending down the latter; much light brown spumous serosity in the trachea.—Lungs and heart healthy.—Pharynx very red; redness suddenly ceasing at the commencement of the œsophagus, which latter presented a healthy appearance.

Stomach: Small quantity of fluid; arborescent redness, but not considerable, of the great cul de sac of the stomach.

All the intestines perfectly healthy.

*Queries.*—1st. May not the nerves be endued with a power of absorption? Yes

2d. May not hydrophobia be produced by nervous absorption? Yes

3d. May not the absorbent power be exceedingly slow, proceeding step by step until the morbific matter arrives at the spinal marrow or brain, when the disease makes its appearance. Yes

It is observed of painters, that, after using the brush a considerable time, a portion of lead constantly insinuating itself between the handle of the brush and the hand, that they become paralytic; but two years of constant exercise in painting is, as I am informed, the earliest period at which that disease manifests itself. This, I submit, cannot be accounted for either by venous or lymphatic absorption.

4th. If the foregoing positions could be proved, would they not lead to this practical result, that, after the bite of a rabid animal, dividing the nerve supplying the part bitten, by cutting off the communication with the nervous origin, would prevent the disease; and if the slowness of nervous absorption could be established, might it not, even a *considerable time after* the bite, by dividing the nerve some distance from and above the part bitten, be still attended by a corresponding beneficial effect? Try it by all means

